# Using a Retro-Reflective Membrane and Laser Doppler Vibrometer for Real-Time Remote Acoustic Sensing and Control

**DOI:** 10.3390/s21113866

**Published:** 2021-06-03

**Authors:** Tong Xiao, Sipei Zhao, Xiaojun Qiu, Benjamin Halkon

**Affiliations:** Centre for Audio, Acoustics and Vibration, University of Technology Sydney, Ultimo, NSW 2007, Australia; Sipei.Zhao@uts.edu.au (S.Z.); Xiaojun.Qiu@uts.edu.au (X.Q.); Benjamin.Halkon@uts.edu.au (B.H.)

**Keywords:** retro-reflective membrane, remote acoustic sensing, sound pressure measurement, laser Doppler vibrometer (LDV), active noise control (ANC)

## Abstract

Microphones have been extensively studied for many decades and their related theories are well-established. However, the physical presence of the sensor itself limits its practicality in many sound field control applications. Laser Doppler vibrometers (LDVs) are commonly used for the remote measurement of surface vibration that are related to the sound field without the introduction of any such physical intervention. This paper investigates the performance and challenges of using a piece of retro-reflective film directly as an acoustic membrane pick-up with an LDV to sense its vibration to form a remote acoustic sensing apparatus. Due to the special properties of the retro-reflective material, the LDV beam can be projected to the target over a wide range of incident angles. Thus, the location of the LDV relative to the pick-up is not severely restricted. This is favourable in many acoustic sensing and control applications. Theoretical analysis and systematic experiments were conducted on the membrane to characterise its performance. One design has been selected for sensing sound pressure level above 20 dB and within the 200 Hz to 4 kHz frequency range. Two example applications—remote speech signal sensing/recording and an active noise control headrest—are presented to demonstrate the benefits of such a remote acoustic sensing apparatus with the retro-reflective material. Particularly, a significant 22.4 dB noise reduction ranging from 300 Hz to 6 kHz has been achieved using the demonstrated active control system. These results demonstrate the potential for such a solution with several key advantages in many applications over traditional microphones, primarily due to its minimal invasiveness.

## 1. Introduction

The performance of some sound field control applications, such as sound field reproduction and active noise control (ANC), is limited by the physical presence of traditional microphones [1,2]. Although the sizes of traditional condenser microphones, including microelectromechanical systems (MEMS) microphones, can be reduced, other associated components (e.g., power supply and data transmission cables) are still required. This is more commanding for fibre-optic microphones [3,4], where the optical fibres carrying the light beams cannot be made wireless.

In ANC headrest systems, for example, the aim is to reduce the sound at a user’s ears with error microphones installed therein. This is not desirable in practice due to their intrusiveness to the user. As the first proposal of such a system [5], it has been one of the main factors limiting the performance. To solve this problem, various virtual sensing algorithms have been proposed to use error microphones placed remote from the user to estimate the sound pressure level (SPL) at the user’s ears [6]. Although such techniques have realised some improvements [7,8], one of the disadvantages is that a large number of microphones are required to control broadband noise originating from various sources. Similar problems also exist in multi-zone sound field control applications [9]. It is, therefore, desirable to develop a remote acoustic sensing system that can cause little intrusion and interference.

A laser Doppler vibrometer (LDV) is a scientific instrument that is used to make non-contact measurements of surface vibration [10,11]. With a very high sensitivity and dynamic range (commercial instruments can measure displacements down to pm (pico-metres) and/or velocities down to nm/s resolution and up to the ’0s m/s peak), it has been widely used in many acoustic and bio-acoustic applications [12,13,14]. In many vibro-acoustic applications, the objects under investigation are present. The vibration levels of these objects can be measured directly by the LDVs for modal analysis and other characterisations [13,15,16]. In many acoustic sensing and control applications, the sound-originating sources can be inaccessible, or the particular point of interest for the pressure measurement is at a space in an air volume. The typical approach is to use one or multiple condenser microphones. However, as mentioned above, the physical presence of the traditional microphones and the cabling imposes various limitations.

In this paper, a piece of retro-reflective tape is directly used as the diaphragm of the remote acoustic sensing arrangement instead of a piece of conventional ultrathin metallised polyester film or metal foil in conventional microphones [2]. One of the benefits is that the tape can be readily excited, which can lead to a wide dynamic range for the solution. Furthermore, the use of the retro-reflective material allows the laser beam to be at a wide range of angles with retained effective performance [17]. More importantly, the medium between the LDV and the sensing retro-reflective membrane is simply air, requiring no physical connection between the physical quantity “pick-up” and the signal conditioning. Many applications can potentially benefit from this configuration, for example, the ANC headrests. So far, as we understand, there have been no comprehensive studies on the use of retro-reflective film as a direct acoustic pressure sensing component when combined with LDVs. Even though the dynamic behaviour of membranes has received significant attentions, this has not yet included retro-reflective material membranes, and the related measured signal quality of LDVs when using them. These characteristics are crucial and must be determined before such pick-ups being used in acoustic sensing and control applications.

The theoretical model of the membrane is firstly reviewed to predict the dynamic behaviour, with experiments being carried out and compared to a traditional condenser microphone as a reference. Practical issues that are unique to the system, such as the incidence location, the incidence angle of the laser beam on the retro-reflective membrane and the delay of the measurement signal due to the alternative signal processing, are systematically investigated through a comprehensive series of rigorous experiments. Two particular examples are presented to further demonstrate the promising applications of the presented apparatus in typical scenarios.

The paper is organised as follows. The theoretical analysis of the vibration of a circular membrane is presented in Section 2. An example of the membrane design is provided in Section 3, accompanied by some discussions of the design. The experimental performance validation in various aspects is reported in Section 4, followed by the practical application challenges presented in Section 5. Some example practical applications and further considerations are presented in Section 6. Conclusions are drawn in Section 7.

## 2. Theoretical Development

**Figure 1:**
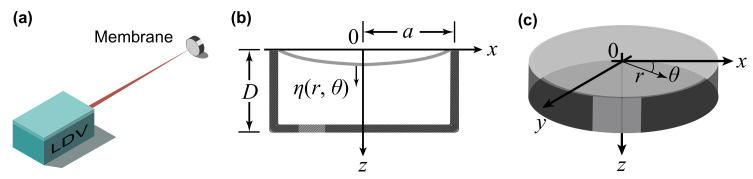
(**a**) Schematic diagram of the demonstrated remote acoustic sensing apparatus, where the LDV measures the acoustically induced vibration of the membrane at the point of testing. (**b**) Cross section of the membrane with a backing cavity. (**c**) Isometric view of the membrane.

The governing equation for a circular membrane shown in Figure 1 can be written as [18,19]

(1) $$\nabla^{2}\eta \left(r,\theta \right)+K^{2}\eta \left(r,\theta \right)=\frac{p_{\mathrm{in}\;}}{T}-\frac{p(z,r,\theta )}{T},$$

where η(r,θ) is the membrane displacement, *r* is the radial coordinate and θ is the azimuthal coordinate. $p_{\mathrm{in}}$ and *p* denote the incident sound pressure and the reaction pressure at the membrane surface, respectively. *K* is the wavenumber of the membrane [20,21] with

(2) $$K=\sqrt{\lambda_{d}^{2}-i\gamma_{d}},\;\lambda_{d}=\omega \sqrt{\frac{\sigma_{M}}{T}},\;\gamma_{d}=\beta \frac{\omega }{T},$$

where i is the complex number, ω is the angular frequency, $\sigma_{M}$ and *T* are the surface density and tension of the membrane, respectively. β is a damping factor.

The vibration of the membrane satisfies the boundary condition at the rim:

(3) $${\left.\eta (r,\theta )\right|}_{r=a}=0,$$

where *a* is the membrane radius. The solution for Equation (1) can be written as

(4) $$\eta \left(r,\theta \right)=\sum_{m=0}^{\infty }\sum_{n=1}^{\infty }A_{mn}\Psi_{mn}\left(r,\theta \right),$$

where $\Psi_{mn}\left(r,\theta \right)$ is the *in vacuo* modal shape of the membrane [18], i.e.,

(5) Ψmn(r,θ)=ReJmKmnrcos(mθ),

where $J_{m}\left(x\right)$ is the *m*-th order Bessel function of the first kind. $K_{mn}$ can be obtained with Equation (2) by using the natural frequency $\omega_{mn}$ determined by the boundary condition ReJmKmna=0.

The modal coefficients $A_{mn}$ in Equation (4) can be obtained as [22]

(6) $$A_{mn}=\frac{1}{T\left(K^{2}-K_{mn}^{2}\right)}\int_{S}\left[p_{\mathrm{in}\;}-p\left(z,r,\theta \right)\right]\Psi_{mn}\left(r,\theta \right)dS,$$

where *S* is the membrane area.

The reaction sound pressure inside the backing cavity is governed by the Helmholtz equation [23],

(7) $$\nabla^{2}p\left(z,r,\theta \right)+k^{2}p\left(z,r,\theta \right)=0,$$

where $k=\omega /c_{0}$ is the acoustic wavenumber with $c_{0}$ being the speed of sound in air. The boundary conditions are

(8a) $$\begin{array}{cc} {\left.\frac{\partial p(z,r,\theta )}{\partial r}\right|}_{r=a} & =0, \end{array}$$

(8b) $$\begin{array}{cc} {\left.\frac{\partial p(z,r,\theta )}{\partial z}\right|}_{z=D} & =0, \end{array}$$

(8c) $$\begin{array}{cc} {\left.\frac{\partial p(z,r,\theta )}{\partial z}\right|}_{z=0} & =-\rho_{0}\omega^{2}\eta , \end{array}$$

where $\rho_{0}$ is the air density, and *D* is the depth of the cavity.

The solution to Equation (7) can be expressed with the modal expansion

(9) $$p\left(z,r,\theta \right)=\sum_{s=0}^{\infty }\sum_{q=1}^{\infty }\left(B_{sq}e^{ik_{z}z}+C_{sq}e^{-ik_{z}z}\right)\Phi_{sq}\left(r,\theta \right),$$

where $k_{z}=\sqrt{k^{2}-k_{sq}^{2}}$, and the modal shape $\Phi_{sq}\left(r,\theta \right)$ can be written as

(10) $$\Phi_{sq}\left(r,\theta \right)=J_{s}\left(kr\right)\cos\left(q\theta \right).$$

By substituting Equation (9) into Equation (8a), one can obtain the eigenvalues $k_{sq}$ from $J_{s}^{\prime }\left(k_{sq}a\right)=0$. In addition, substituting Equation (9) into Equations (8b) and (8c) yields, respectively,

(11) $$B_{sq}e^{ik_{z}D}-C_{sq}e^{-ik_{z}D}=0,$$

(12) $$ik_{z}\left(B_{sq}-C_{sq}\right)\Phi_{sq}\left(r,\theta \right)=-\rho_{0}\omega^{2}\eta .$$

Applying Equation (9) into Equation (6) with z=0 and integrating over the membrane surface derives

(13) $$T\left(K^{2}-K_{mn}^{2}\right)A_{mn}+L_{mn,sq}\left(1+e^{2ik_{z}D}\right)B_{sq}=P_{\mathrm{in},mn}\;,$$

where Equation (11) is used to eliminate $C_{sq}$, $L_{sq,mn}$ denotes the coupling between the membrane vibration and the sound pressure inside the cavity [23], which can be expressed as

(14) Lmn,sq=∫SΦsq(r,θ)Ψmn(r,θ)dS=Re2πa·δms·εmsksq−KmnKmnJsksqaJm−1Kmna−ksqJmKmnaJs−1ksqa,

where $\delta_{ms}$ is 1 for m=s and 0 for m≠s, $\varepsilon_{ms}$ is 1 for m=n=0 and 0.5 for m=s≠0.

The (m,n)th mode modal coefficient of the incident sound pressure at the membrane surface in Equation (13) can be written as

(15) $$P_{\mathrm{in}\;,mn}=\int_{S}p_{\mathrm{in}\;}\Psi_{mn}\left(r,\theta \right)dS=\left\{\begin{array}{cc} \frac{2\pi ap_{\mathrm{in}\;}}{K_{0n}}J_{1}\left(K_{0n}a\right), & m=0\\ 0, & m\neq 0 \end{array}\right.,$$

where the incident sound pressure $p_{\mathrm{in}}$ is assumed to be uniform over the membrane surface.

Similarly, substituting Equation (4) into Equation (12) yields

(16) $$\rho_{0}\omega^{2}\sum_{m=0}^{\infty }\sum_{n=1}^{\infty }A_{mn}\Psi_{mn}\left(r,\theta \right)+ik_{z}\left(1-e^{2ik_{z}D}\right)\Phi_{sq}\left(r,\theta \right)B_{sq}=0.$$

Multiplying $\Psi_{mn}\left(r,\theta \right)$ on both sides of Equation (16) and integrating over the membrane surface generates

(17) $$\pi a^{2}\rho_{0}\omega^{2}\Lambda_{mn}A_{mn}+ik_{z}\left(1-e^{2ik_{z}D}\right)L_{mn,sq}B_{sq}=0,$$

where $\Lambda_{0n}={\left[J_{1}\left(K_{0n}a\right)\right]}^{2}$ and $\Lambda_{mn}=1/2{\left[J_{m-1}\left(K_{mn}a\right)\right]}^{2}$ for m>0.

By solving Equations (13) and (17) simultaneously, the modal coefficients $A_{mn}$ and $B_{sq}$ can be obtained, and $C_{sq}$ can then be derived from $B_{sq}$ in Equation (11). After the modal coefficients are derived, both the membrane displacement and sound pressure inside the cavity can be calculated from Equations (4) and (9), respectively. As the wavelength for the sound pressure in the frequency range of interest (up to 4 kHz) is much larger than the size of the membrane and the cavity, both the membrane vibration and sound pressure inside the cavity are assumed to be axisymmetric, which means the subscript *m* should be 0 in all the above equations.

## 3. Design and Numerical Modelling

**Figure 2:**
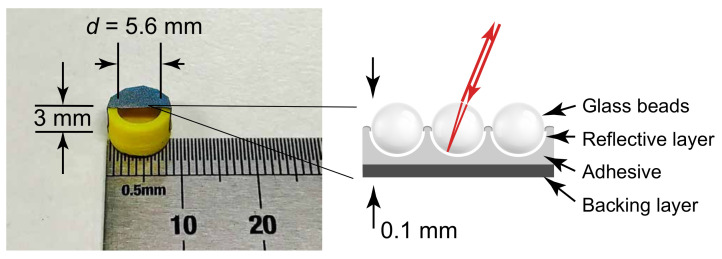
Membrane specimen (including the backing cavity) with a piece of retro-reflective film (half shown here for illustrating the backing cavity) and its composition; detailed specifications provided in Table 1.

Figure 2 shows an example of the illustrated membrane specimen, where a piece of retro-reflective film (half shown for illustration purpose) had been applied upon a cylindrical enclosed tube (inner diameter = 5.6 mm, depth of the backing cavity = 3 mm) made of 3D printed acrylonitrile butadiene styrene. The commercial retro-reflective film (3M—Scotchlite Sheeting 7610 [17]) was pre-coated with glass beads and a reflective layer to ensure that the reflected laser illumination can be directed in the same direction as in the inbound path back to the LDV optics. The thickness of the retro-reflective film was about 0.1 mm, with the size of the glass beads about 40 μm. The material of the supporting base tube can be either polymeric or metallic, even though polymeric material is preferable in certain applications, such as near a magnetic resonance imaging machine, or in applications requiring the membrane to be lightweight. When the membrane was stretched over the supporting base, it can be either clamped by an outer ring as in conventional condenser microphones [24], or adhered to the side of the supporting base tube. The latter design was chosen here for simplified construction as shown in Figure 2. Table 1 provides the parameters of the membrane specimen with its backing cavity.

**Figure 3:**
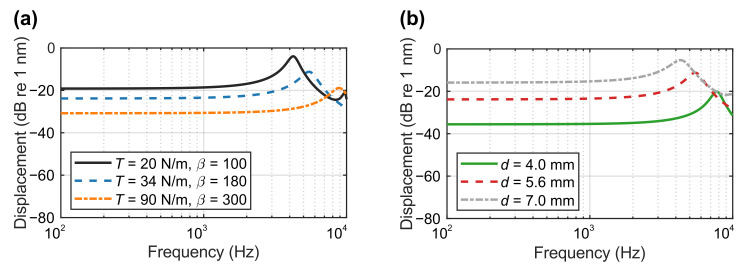
The analytical displacements of the membrane (**a**) with three tension values and the adjusted damping factors, and (**b**) with three diameter values.

Using the parameters in Table 1, the displacement of the membrane specimen excited by a broadband white noise can be calculated using the analytical solution provided in Section 2. Three values of the membrane tension *T* at 20 N/m, 34 N/m and 90 N/m were investigated with a corresponding damping factor β of 100, 180 and 300, retrospectively, selected. The diameter of the membrane was 5.6 mm in this case. The resulting displacements are compared in Figure 3a. For *T* = 20 N/m, the vibration resonance occurred at about 4.2 kHz. Although the overall level was the highest among the three examples when subject to the same SPL, i.e., 1 Pa, the working frequency range was only below 2 kHz, which cannot meet requirements in many applications. When *T* = 90 N/m; however, the resonant frequency occurred at approximately 9 kHz. Although the working frequency range was wider (up to 6 kHz), the overall level was low, indicating a low sensitivity that is not desirable in some situations. When *T* = 34 N/m, the resonance occurred at 5.5 kHz, allowing the working frequency range of the demonstrated apparatus to be up to approximately 4 kHz. Meanwhile, the overall level (i.e., the sensitivity) was not significantly reduced. Therefore, it was reasonable to choose the design when the tension of the membrane is 34 N/m with a damping factor of 180.

In addition, the diameter of the membrane *d* was considered. Figure 3b shows three examples when the diameter is 4.0 mm, 5.6 mm and 7.0 mm. The tension and the damping factor remained the same at 34 N/m and 180, respectively. Similar to the case of the membrane tension, when *d* = 4.0 mm, the vibration resonance was at 7.8 kHz, but the sensitivity was significantly low. On the other hand, when *d* = 7.0 mm, the sensitivity was increased; however, the resonance decreased to 4.3 kHz, leaving a working frequency range below 3 kHz only. When *d* = 5.6 mm, the working frequency range was up to 4 kHz with a satisfactory sensitivity compared to other scenarios.

In summary, the membrane tension is an important design factor. Increasing the membrane tension, decreasing the diameter or decreasing the surface density can increase the resonance frequency, thus extending the working frequency range. However, the sensitivity and the dynamic range are reduced correspondingly [18]. Therefore, for a given microphone design and material, i.e., a fixed diameter and surface density, this retro-reflective membrane should be properly stretched to have a balance between a sufficient sensitivity and a high natural frequency for specified applications.

With the frequency range of the membrane demonstrated hereinafter, the parameters of the retro-reflective membrane were chosen as *d* = 5.6 mm, *T* = 34 N/m and β = 180 such that the presented remote sensing system can measure various types of sound and noise, including human speech, which is generally below 4 kHz.

## 4. Experimental Performance Validation

**Figure 4:**
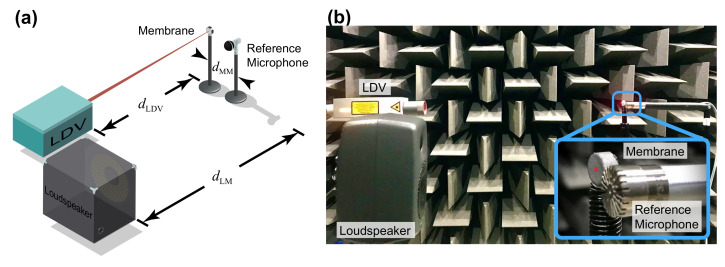
(**a**) System configuration. (**b**) Experimental setup.

As shown in Section 2, the displacement of the membrane is proportional to the incident sound pressure, and the output signal from the LDV can be multiplied by the sensitivity, converting the electrical signal from the LDV to the corresponding sound pressure. This is a similar approach to that used for condenser microphones, which use a sensitivity to convert the measured capacitance (converted to voltage) into sound pressure. As the sound pressure at the exact point of the membrane is unknown, a reference microphone (or multiple microphones) can be placed extremely close to the membrane specimen to monitor the sound pressure for calibration. Figure 4a shows the system configuration for calibration and evaluation. The experimental setup, shown in Figure 4b, was carried out in a hemi-anechoic chamber. The membrane under test was placed on a metal rod with an adjacent reference microphone (Brüel & Kjær Type 4191) placed a distance of 0.01 m ($d_{\mathrm{MM}}$) away. The LDV (Polytec NLV-2500-5) was located approximately 1.3 m ($d_{\mathrm{LDV}}$) away with a displacement sensitivity setting of 50 nm/V. The laser beam was maintained at the centre of the membrane throughout the experiments. A loudspeaker (Genelec 8010A) was placed approximately 1.0 m ($d_{\mathrm{LM}}$) from the microphone. The SPL measured by the reference microphone was 90 dB.

**Figure 5:**
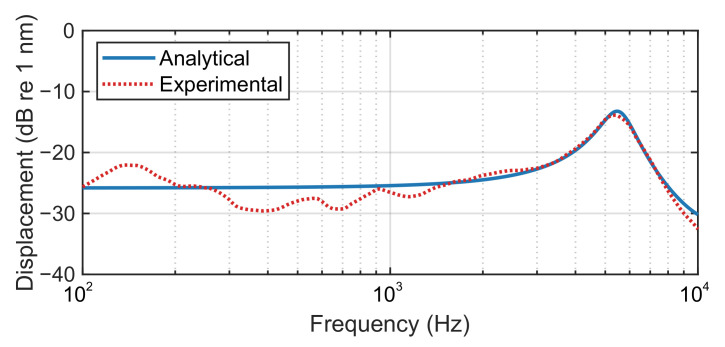
Analytical and experimental spectrum of the displacement of a membrane specimen obtained with white noise excitation, where a resonance occurs at about 5.5 kHz.

The spectrum of the membrane displacement was obtained initially and compared with the analytical solution in Section 2 in Figure 5. It can be seen that the trend of the experimental results generally agrees with the analytical equivalent. There is a little discrepancy in the low frequency range, which might be caused by some minor distortion in the membrane and some minor reflections from the hemi-anechoic chamber. Nonetheless, the analytical model is valid for predicting the behaviour of the membrane.

### 4.1. Sensitivity

**Figure 6:**
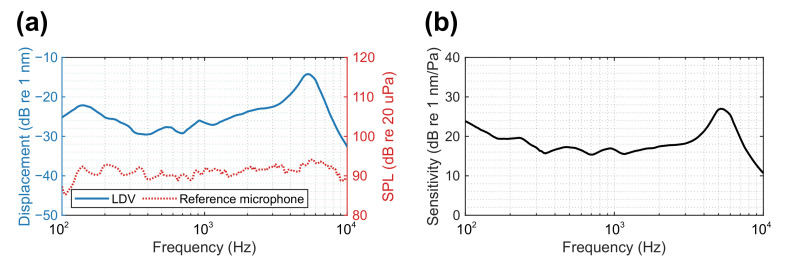
(**a**) Spectrum of the membrane displacement from the LDV and the SPL by the reference microphone and (**b**) the corresponding sensitivity of the membrane as in displacement with respect to SPL monitored by the reference microphone.

Conventional microphones use capacitance to represent the displacement of the microphone diaphragm, whereas the remote acoustic sensing apparatus uses the LDV to measure the membrane vibration displacement directly. The unit of sensitivity becomes nm/Pa instead of mV/Pa as for conventional microphones. However, the sensitivity of the proposed sensor cannot be made exactly like that of a condenser microphone. The sensitivity can be calculated by comparing the membrane displacement from the LDV to the SPL from the adjacent reference microphone. Figure 6 shows the both the displacement measurement from the LDV and the SPL measured by the reference microphone and the signals were recorded using the “Steady-State Response (SSR) Analyzer” in the Brüel & Kjær Pulse LabShop software. The sensitivity of the membrane was calculated from the difference between the two as about 6.7 nm/Pa (or 16.6 dB re 1 nm/Pa) for the range of 200 Hz to 4 kHz.

Notice that the previous fluctuations in the displacement measurement is also present in the reference microphone, which can confirm some minor reflections in the testing environment. When the sensitivity was calculated, the fluctuation is reduced to about ±2 dB from 200 Hz to about 4 kHz. However, the magnitude of the sensitivity is still high below 200 Hz, e.g., about 7 dB difference at 100 Hz, even though the displacement measurement from Figure 5 was in agreement with the analytical result. This may be caused by the sensor head vibration from the LDV, which is more likely excited by low frequency sound. This issue has been extensively studied and solutions can be found in [25].

### 4.2. Noise Floor and Limits

**Figure 7:**
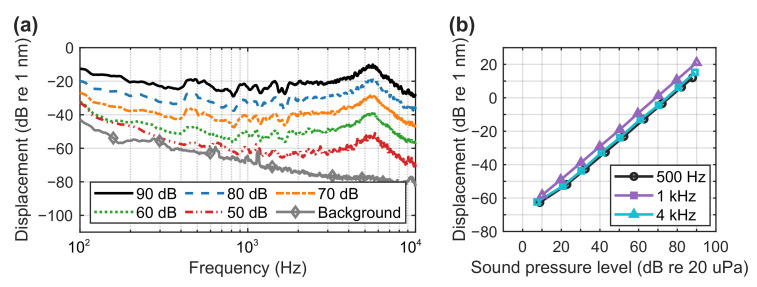
Membrane displacement measurements with different SPLs from (**a**) an overall value of 90 dB to 50 dB and the background noise of the LDV, and (**b**) 90 dB to 10 dB measured by a reference microphone at 500 Hz, 1 kHz and 4 kHz.

The overall background sound pressure level in the hemi-anechoic chamber measured by the reference microphone was approximately 23 dBA to 20 μPa. Meanwhile, the total background noise of the LDV was measured to be about −51 dBA re 1 nm, which was determined by pointing the LDV to the floor of the hemi-anechoic chamber. The background noise of the LDV cannot be simply translated to a sound pressure level using the sensitivity because the membrane displacement may not be proportional at a very low sound pressure level. To investigate this, the sound source with a white noise was decreased from a total value of 90 dB to 50 dB with a step of 10 dB. As shown in Figure 7a, the decrements were mostly consistent across the spectrum. In addition, three tonal signals at 500 Hz, 1 kHz and 4 kHz were set to various levels from 90 dB to 10 dB to observe the corresponding membrane displacement, which is shown in Figure 7b. Overall, the membrane displacement is valid only when the SPL is above an overall level of about 60 dB. Below this level, the change in the membrane displacement is not proportional to the one in the SPL. This can also be observed in Figure 7, where the decrements of the displacement from 20 dB to 10 dB below 300 Hz did not follow the trend. To measure an even lower SPL, a larger membrane can be used, though the effective frequency range may be reduced as discussed in Section 3. The maximum SPL in these tests was only up to 90 dB due to the rating of the sound source (96 dB maximum). However, the upper limit of the proposed system could be well beyond this, likely above 130 dB.

### 4.3. Total Harmonic Distortion (THD)

**Figure 8:**
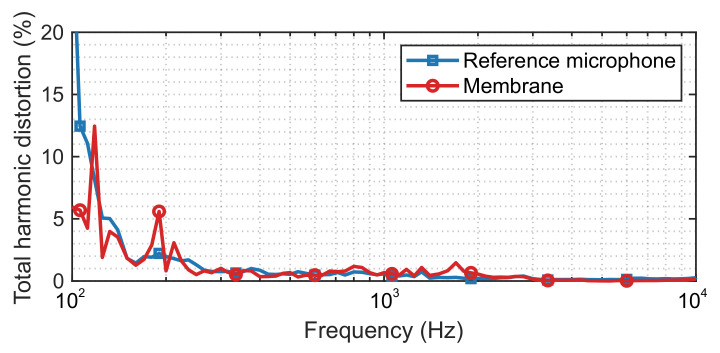
THD spectrum obtained by the reference microphone and the membrane specimen.

The harmonic distortion is an important property to evaluate a microphone for sound measurements. Because the LDV measures the vibration of the target, the main sources contributing to the distortion are anticipated to be the membrane itself and the supporting base. The total harmonic distortion (THD) at a frequency can be calculated with

(18) $$\mathrm{THD}=\sqrt{\frac{A_{2}^{2}+A_{3}^{2}+A_{4}^{2}+A_{5}^{2}}{A_{1}^{2}+A_{2}^{2}+A_{3}^{2}+A_{4}^{2}+A_{5}^{2}}}\times 100,$$

where $A_{j}$ is the amplitude of the *j*-th harmonic. The THD spectrum measured by the reference microphone and the constructed membrane is shown in Figure 8. As a well-designed and manufactured sensor, the THD of the reference microphone is small overall, which is below 3% above 150 Hz and below 1% above 300 Hz. The custom-made retro-reflective membrane has a similar result, except for some slightly higher THD at a few frequencies below 300 Hz. Above 300 Hz, the THD for the custom-made retro-reflective membrane is also approximately 1%.

## 5. Practical Application Associated Challenges

### 5.1. Laser Beam Incidence Location

**Figure 9:**
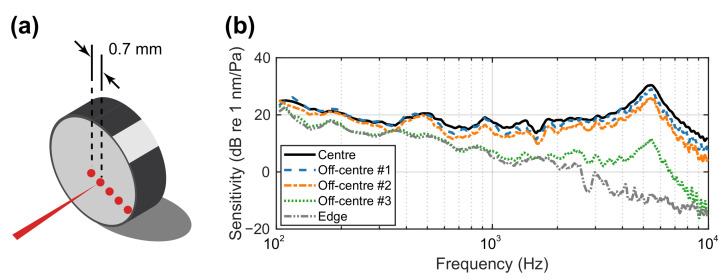
(**a**) Five evaluation points for the laser beam on the membrane specimen-centre, off-centre #1, off-centre #2 and the edge. The interval of the points is about 0.7 mm. (**b**) Sensitivity of the membrane specimen at the five points.

The circular membrane displacement η(r,θ) is related to the radial distance *r*, which is expected to be the most significant at the centre of the membrane. When the laser beam projection point on the membrane deviates from the centre, the detected vibration level drops. The vibration level at the edge should ideally approach zero. However, due to the lightweight nature of the structure, the boundary is not completely free of vibration, particularly in the low-frequency range. Figure 9 shows the experimental results, where five laser beam incidence points on the membrane from the centre to the edge, each separated by 0.7 mm, are shown. The membrane vibration level drops significantly in the higher frequency range for increased distance from the centre. For example, at 5.5 kHz, the sensitivity of the sensor dropped from about 32.7 dB at the centre to about 31.3 dB, 28.1 dB, 14.2 dB and −7.0 dB at off-centre points #1, #2, #3 and at the edge, respectively. Sensitivity is also reduced in the low-frequency range but by a diminished amount. This is primarily caused by the vibration from the whole structure, which mostly occurs at the low frequencies. If any deviation occurs in the application, adjustment should be made to equalise the measurement.

### 5.2. Laser Beam Incidence Angle

**Figure 10:**
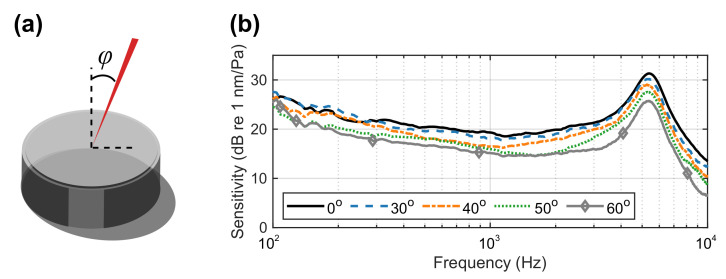
(**a**) Incidence of the laser beam with an angle φ. (**b**) Sensitivity of the membrane specimen with different incoming angles.

LDVs have been used for many instruments with polished metallic surfaces, for example, a microphone diaphragm [13,26]. However, these mirror-like surfaces only allow the laser beam incidence to be in the normal direction. By using retro-reflective film as the membrane, the laser beam from the LDV can be projected onto the membrane from a wide range of non-normal incidence angles. Figure 10 presents the membrane vibration levels when the laser beam from the LDV was kept at the centre but with various incidence angles. When in the normal direction, the measured membrane vibration level was the most significant. As the incidence angle increases, the overall vibration level reduces. The magnitude of the displacement should be applied with cos(φ) such that it has the most significant value when φ=0, that is, the normal direction; it has the least significant value when φ=90, that is, the laser beam is parallel with the membrane. For example, at 5.5 kHz, the magnitude was approximately 32.4 dB. When the incident angles are at 10 and 20 degrees, there was no obvious difference, as the difference, in theory, is less than 1 dB. When the incidence is at 30 degrees, the magnitude reduced to 31.5 dB. At 40, 50 and 60 degrees, the magnitude dropped to 31.0 dB, 29.0 dB and 27.7 dB, respectively. This reduction follows the expectation. When the incidence is below 30 degrees, the reduction in the magnitude is less than 1 dB, which is not large to cause significant errors. When the incidence is beyond 30 degrees, the reduction in the magnitude cannot be neglected and adjustments must be made for correct measurements.

### 5.3. Signal Delay

Due to the electronic signal conditioning components within the LDV, delay of the measured signal should be investigated. With the configuration and setup shown in Figure 4, signals from the reference microphone and the LDV were recorded synchronously. The distances from the loudspeaker to the membrane and the reference microphone are the same because they are close to each other. Thus, the acoustical transmission delays for the two sensors are the same. By only comparing the displacement signal from the LDV (Polytec NLV-2500-5) with the SPL measurement from the reference microphone, it was found that there was only 3-sample delay when the sampling rate of the recorded data was set to 65.536 kHz, i.e., there is about 46 μs delay of the LDV signal with respect to microphone signal.

## 6. Example Practical Applications and Further Considerations

### 6.1. Remote Speech Recording/Measurement

**Figure 11:**
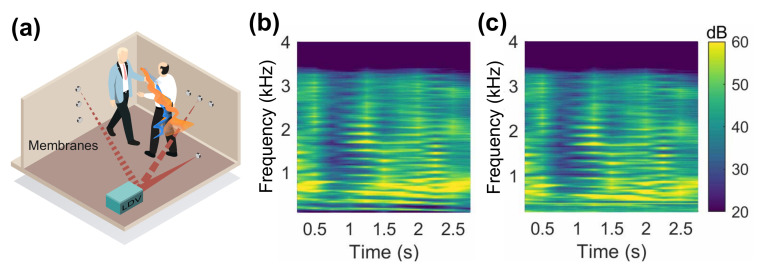
(**a**) Illustration of the remote speech recording system. (**b**) Spectrogram of a speech signal recorded by a microphone. (**c**) Spectrogram of the same speech signal recorded by the demonstrated acoustic sensing apparatus.

One example is shown in Figure 11 where the demonstrated acoustic sensing apparatus with a retro-reflective membrane is used for the recording of a speech signal. The loudspeaker generated a 3 s speech signal (see “speech.mat” in [27]), which was recorded by both the microphone and the proposed system simultaneously. The results from the two instruments are fairly similar, except for some minor discrepancies at approximately 100 Hz, which could be due to the interference from the supporting base at the low frequencies. Nonetheless, the speech information by the proposed apparatus is preserved well overall compared to a professional measurement microphone. Thus, it can be favourable for a remote sound measurement system which requires easy and light installation without cables and fast data transmission.

### 6.2. Active Noise Control (ANC)

The acoustic sensing apparatus provides an alternative method when commonly used condenser microphones cannot be installed or used. For example, ANC headrest can take advantage of such a system to improve the control performance without causing large disturbance to the user [1,6,7,8]. Under some circumstances, it is not desirable for people to wear noise-cancelling headphones or earmuffs, ANC headrests can be used where the secondary (control) loudspeakers are placed at the headrest of a chair to create a quiet zone around the user’s ears by interfering destructively with the primary (undesirable) noise. In order to have a satisfactory control performance, the noise going into the user’s ears (used as the error signal) needs to be determined preferably without installing microphones in the user’s ears. Although the size of various microphones, including the MEMS microphones, can be small, other associated componentry (e.g., power supply, signal conditioning and data transmission cables) are still required [28]. With two membranes described in this paper placed in the user’s ears, the LDV can determine the acoustic information remotely without any physical connections [29,30].

**Figure 12:**
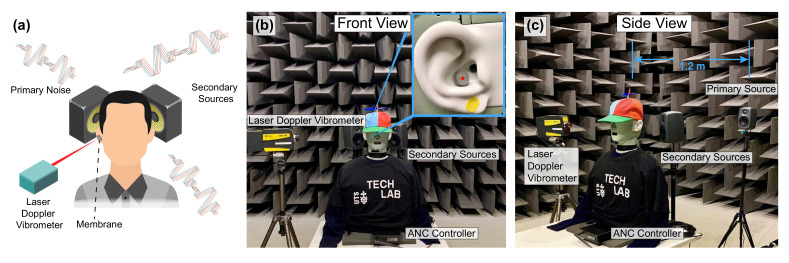
(**a**) Schematic of the presented acoustic sensing apparatus used for ANC where a retro-reflective membrane is placed in one of the user’s ears and the LDV measures the vibration of the membrane as the error signal. (**b**) Front view and (**c**) side view of the experimental setup.

A simple schematic and the corresponding experimental setup of such a system is shown in Figure 12. For demonstration purpose, a head and torso simulator (HATS, Brüel & Kjær Type 4128-C) equipped with two ear simulators were used to evaluate the control performance. The LDV used in this system was the Polytec PDV-100 Portable Digital Vibrometer. The membrane used in this system has a similar design to the one shown in Figure 2, except that the inner diameter in this case was about 8.2 mm. The controller used was an Antysound TigerANC WIFI-Q in which the robust and effective Filtered-reference Least Mean Square (FXLMS) algorithm [1] is used. Note that the error signal in this case is that from the LDV rather than from a conventional microphone as would commonly be the case in this other such sound field control systems.

**Figure 13:**
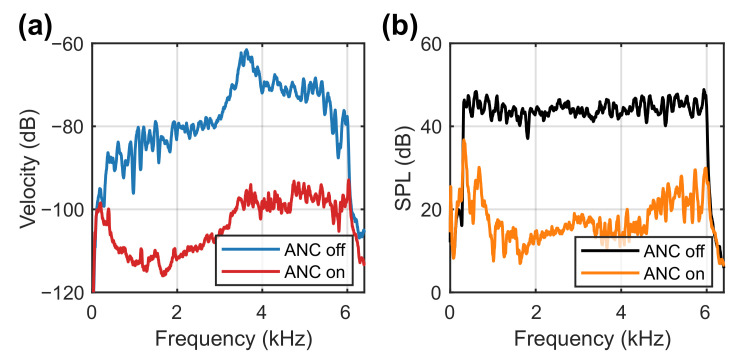
Spectra of (**a**) the membrane velocity and (**b**) the corresponding sound pressure level (dB re 20 μPa) observed by the HATS without and with ANC for a grey noise from 300 Hz to 6 kHz.

The control performance obtained from one ear simulator of the HATS for a grey noise (300 Hz–6 kHz) is shown in Figure 13. The membrane velocity with ANC off and on is illustrated in Figure 13a, where it was controlled by 28.3 dB from an overall value of −38.8 dB to −67.1 dB. The SPL observed by the HATS is shown in Figure 13b, where it was controlled by 22.4 dB from 77.0 dB to 54.6 dB. Compared to the noise reduction of about 15 dB in [29], the reduction level is more significant. This is due to the background noise. In this case, the hemi-anechoic chamber has a background noise of about 23.0 dBA, which is noticeably lower than the 38.5 dBA in [29].

The state-of-the-art system [8] used four remote microphones to estimate the sound at the user’s ears coming from one direction, where the upper limit of the controlled noise was up to 1 kHz. To achieve the performance shown in Figure 13, the number of monitoring microphones installed around the user has to be increased by several times. In addition, in order to control the noise coming from multiple directions, the required number of monitoring microphones needs to be increased even further. In contrast, the presented acoustic sensing apparatus can directly determine the acoustic information at the user’s ear canal for ANC.

**Figure 14:**
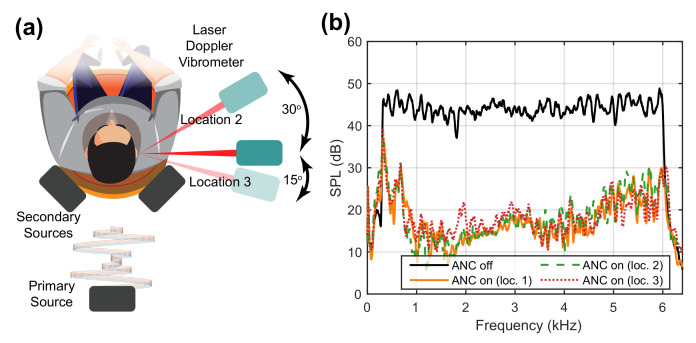
(**a**) Illustration of the alternative two locations for the LDV. (**b**) The sound pressure reduction levels observed by the HATS for the grey noise (300 Hz–6 kHz) at three locations.

The result shown above was obtained by randomly placing the LDV at the side (about $90^{\circ }$) of the HATS. The advantage of using the retro-reflective material as the membrane is that the laser beam from the LDV can be projected from a wide range of angles. To examine this, the LDV was placed at another two locations as shown in Figure 14, and the corresponding SPLs observed by the HATS are depicted in Figure 14b. The control performances with the laser beam from another two angles remained the same as before. Therefore, using the retro-reflective material makes it possible to maintain the control performance if there are any movements from the user. In practice, a head movement tracking system needs to be used. A pair of orthogonally placed galvanometer-driven mirrors could be added to steer the laser beam, associated with a camera-based head-tracking system. Thus, the LDV can keep measuring the vibration of the membrane even when the membrane (i.e., the user’s head) moves. A simple head tracking system for illustrating purpose was demonstrated in [29].

## 7. Conclusions

This paper reports a remote acoustic sensing apparatus that consists of a retro-reflective membrane and an LDV at a remote location. This system can measure acoustical pressure signals at remote locations with a minimal instrumentation footprint. The small circular membrane pick-up without any cables can be placed at the location of interest, whereby an LDV can then measure the acoustically induced membrane surface vibration at that remote location. The membrane was purposefully chosen to be retro-reflective to allow a wide range of LDV laser beam incidence angles. The sensitivity of the proposed sensor was calculated, and the noise floor of the LDV and the THD are reported. In addition, some practical issues in applications, such as the exact laser beam incidence location on the membrane, the incidence angle and the signal latency, were addressed. The demonstrated membrane has a working frequency range from 200 Hz to 4 kHz, which is sufficient in some acoustic sensing, speech measuring applications. The initial measurement results indicate that the presented acoustic sensing apparatus has potential in applications when traditional microphones cannot be installed, such as in an ANC headrest. In this case, the frequency range of the controlled noise was from 300 Hz to 6 kHz. The sound pressure reduction level was over 20 dB.

However, it should be noted that there are some factors potentially limiting the functionality of the proposed system. First, although the system allows for remote and wireless acoustic measurements, small membranes are still required as the acoustic pick-ups. Second, since the measurements are made from the LDVs placed at a remote location, the paths for the laser beams must be cleared and thus the placement of these devices can be limited. One possible solution can be to include one or multiple small galvanometer-driven beam-steering mirrors to redirect the laser beams depending on a specific application. Last, the laser beams from LDVs might be disturbing or hazardous particularly when human users are involved. Although eye-safe, such a system should be designed such that these hazards shall be minimised.

Future work will include designing the membrane to yield a solution with a wider frequency range. For example, using a thinner material (e.g., graphene) treated to be retro-reflective can possibly be used to both broaden the frequency range and improve the sensitivity.

## Figures and Tables

**Table 1:** Parameters of the designed membrane specimen with the backing cavity.

Quantity		Symbol	Unit	Value
Membrane Specimen (including the backing cavity)	Diameter	*d*	mm	4.0, 5.6, 7.0
	Thickness	*h*	mm	0.1
	Density	$\rho_{M}$	$\mathrm{kg}/m^{3}$	204
	Tension *	*T*	N/m	20, 34, 90
	Damping factor	β	–	100, 180, 300
	Cavity depth	*D*	mm	3
Air	Sound speed	$c_{0}$	m/s	340
	Density	$\rho_{0}$	$\mathrm{kg}/m^{3}$	1.205

## Data Availability

The data that supports the findings of this study are available from the authors on reasonable request, see author contributions for specific data sets.

## Abbreviations

The following abbreviations are used in this manuscript:

ANC	Active Noise Control
FXLMS	Filtered-reference Least Mean Square
HATS	Head And Torso Simulator
LDV	Laser Doppler Vibrometer
MEMS	Microelectromechanical Systems
SPL	Sound Pressure Level
THD	Total Harmonic Distortion
